# Serum and cerebrospinal fluid biomarkers for risk stratification of hospitalized neonates and young infants with suspected serious bacterial infections: a secondary analysis of a prospective cohort study

**DOI:** 10.3389/fimmu.2026.1873954

**Published:** 2026-10-16

**Authors:** Chaymae El Abbass, Luis Alberto Benegas, Andrea Alemany, Cristina Garcia-Mauriño, Ilham Elouardighi, Imane Zizi, Najat Amalik, Lamya El Laziji, Sobha El Ftouh, Rachid Babi, Khalid Bounagua, Nouhayla Bouali, Sara Ajanovic, Sara Arias, Dan Ouchi, Fio Vialard, David Torres-Fernandez, Pere Millat-Martinez, Diana Barrios, Rosauro Varo, Kevin C. Kain, Ruth Aguilar, Carlota Dobaño, Gemma Moncunill, Houssain Tligui, Bàrbara Baro, Quique Bassat, Amina Barkat

**Affiliations:** 1 Research Team on Health and Nutrition of Mother and Child, Laboratory of Biostatistics, Clinical Research and Epidemiology, Faculty of Medicine and Pharmacy, Mohammed V University in Rabat, Rabat, Morocco; 2 National Reference Center for Neonatology and Nutrition, Children’s Hospital, Ibn Sina University Hospital Centre, Rabat, Morocco; 3 ISGlobal, Barcelona, Spain; 4 Department of Preventive Medicine and Epidemiology, Hospital Clínic, Barcelona, Spain; 5 Research Laboratory of Ibn Sina University Hospital Centre - Faculty of Medicine and Pharmacy, Mohammed V University in Rabat, Rabat, Morocco; 6 Mohammed VI International School of Public Health, Mohammed VI University of Sciences and Health (UM6SS), Casablanca, Morocco; 7 Facultat de Medicina i Ciències de la Salut, Universitat de Barcelona (UB), Barcelona, Spain; 8 Centro de Investigação em Saúde de Manhiça (CISM), Maputo, Mozambique; 9 Pediatric Infectious Diseases and Immunodeficiencies Unit, Children’s Hospital, Vall d’Hebron Barcelona Hospital Campus, Barcelona, Spain; 10 Infection and Immunity in Pediatric Patients, Vall d’Hebron Research Institute, Barcelona, Spain; 11 Institut de Recerca Sant Joan de Deu, Hospital Sant Joan de Deu, Barcelona, Spain; 12 CIBER de Epidemiología y Salud Pública, Instituto de Salud Carlos III, Madrid, Spain; 13 Tropical Disease Unit, University of Toronto, UHN- Toronto General Hospital, Toronto, ON, Canada; 14 CIBER Enfermedades Infecciosas (CIBERINFEC), Instituto de Salud Carlos III, Madrid, Spain; 15 ICREA, Barcelona, Spain; 16 Pediatrics Department, Hospital Sant Joan de Déu, Universitat de Barcelona, Esplugues, Barcelona, Spain; 17 Institut Clínic de Medicina i Dermatologia, Hospital Clínic de Barcelona, Barcelona, Spain

**Keywords:** biomarkers, host response, meningitis, neonates, sepsis, severe infections

## Abstract

**Background:**

Improved risk stratification strategies for neonates and young infants are urgently needed to reduce the substantial mortality burden associated with infectious diseases, particularly in low- and middle-income settings. Evidence supporting the use of host-response biomarkers to predict disease severity in this population remains limited.

**Methods:**

We conducted a secondary analysis of a prospective cohort study at a National Reference Neonatology Centre in Morocco and evaluated the prognostic performance of host-response biomarkers measured in serum and cerebrospinal fluid (CSF) to predict in-hospital mortality among hospitalized sick neonates and young infants (<2 months). Twenty-one biomarkers of immune activation, endothelial dysfunction, and neuronal injury were measured using Luminex and compared head-to-head.

**Results:**

Among 179 participants with available serum and/or CSF samples included in this analysis, 9% died during hospitalization (11/136 in the serum sample set, and 12/148 in the CSF sample set). In serum, lower concentrations of TRAIL and higher concentrations of Angpt-2, IL-8, and uPAR, were associated with in-hospital death, with TRAIL showing the strongest prognostic performance (AUROC 0.86; 95% CI 0.78-0.93). In CSF, PCT was the strongest predictor of severe progression for in-hospital death (AUROC 0.87; 95% CI 0.80-0.95), whereas higher levels of IL-6, IL-12p70, TNFR1, TREM-1, Angpt-1, NGAL, and NSE were associated with higher odds of probable or laboratory-confirmed meningitis.

**Discussion:**

Serum levels of TRAIL showed an exploratory association with disease severity in hospitalized neonates and young infants with suspected serious bacterial infections. Larger multicenter studies are needed to validate these findings using multiple biological matrices, characterize kinetics, and determine whether these markers may have clinical utility for risk stratification in this vulnerable population.

## Introduction

Neonates and young infants are particularly vulnerable to serious infectious diseases. Globally, over half a million infants die each year due to the leading serious bacterial infections (SBIs) - sepsis, meningitis, and pneumonia (1) - with the burden of neonatal sepsis and associated mortality falling disproportionately on low- and middle-income countries (LMICs) (2–4). Diagnosis and management of SBIs in this population are particularly challenging because of nonspecific clinical signs (1, 5–8), difficulties in specimen collection (9), rapid disease progression, and limited accuracy of laboratory tests (10), barriers that are further exacerbated in LMIC. Notably, up to 84% of infection-related newborn deaths could be prevented with earlier detection and timely treatment (2, 11).

Immune and endothelial activation markers have demonstrated strong value for predicting disease severity across a broad spectrum of infectious diseases, both in adults and older children (12–20). Growing interest has focused on integrating such biomarkers with clinical scores (1, 21, 22), to enhance early identification of patients most likely to benefit from targeted treatments and closer monitoring. In neonates and young infants, biomarker research has largely focused on the diagnosis of neonatal sepsis, identifying promising candidates such as procalcitonin (PCT), soluble triggering receptor expressed on myeloid cells-1 (sTREM-1), interleukin-6 (IL-6), and interferon gamma-induced protein 10 (IP-10) (23–30). Interest has also extended to biomarkers in pregnant women colonized with relevant pathogens in LMIC to predict neonatal outcomes, with studies reporting associations between maternal IL-17 and IL-6 and perinatal transmission of group B streptococcus, as well as *E. Coli* and *K. pneumoniae*, respectively (31–33).

By contrast, evidence supporting biomarkers to predict severity and mortality in neonates and young infants remains limited and inconsistent. Although a few markers, including C-reactive protein (CRP), PCT, IL-6, angiopoietin-2 (Angpt-2), IL-10, and sTREM-1 have shown promising results (34–42), most studies have been small, heterogeneous in design and sample type, and predominantly conducted in high-income settings. There is therefore an urgent need to identify robust biomarkers that can support evidence-based, point-of-care risk stratification of neonates and young infants, particularly in LMICs, where neonatal mortality is highest and resources are scarce.

The main aim of this study was to evaluate a wide panel of biomarkers measured in serum and CSF of neonates and young infants hospitalized with suspected SBIs in order to find good predictors of progression to in-hospital death.

## Methods

### Study design and population

This study is a prespecified secondary single-center analysis of the UNITED-Meningitis project, a prospective multicountry cohort study designed to validate a portable non-invasive device for the rapid screening of meningeal infections in neonates and infants (43). The present analysis includes participants recruited at the National Reference Centre for Neonatology and Nutrition of the Children’s Hospital, Ibn Sina University Hospital Centre, Rabat. Recruitment was conducted between 17^th^ March 2022 and 10^th^ January 2024. The study was approved by national and institutional ethics committees in Morocco and Spain, including the Faculty of Medicine and Pharmacy of Rabat (C70-20), and Hospital Clínic in Barcelona (HCB/2022/0300). Written informed consent was obtained before enrolment.

Eligible participants were hospitalized neonates and young infants ≤2 months with SBI and a clinical indication for lumbar puncture (LP), defined by neurological symptoms, or increased inflammatory markers (CRP≥20 mg/L or PCT >0.5 ng/mL). Infants with conditions contraindicating LP were excluded. Participants were excluded from this secondary analysis only when the residual sample volume was insufficient for biomarker quantification, reflecting the challenges of obtaining adequate blood and CSF volumes in sick neonates.

### Study procedures

All data were prospectively collected using standardized electronic case report forms, including baseline perinatal and clinical characteristics, LP-related variables, laboratory results, and clinical outcomes from admission to discharge. Blood and CSF samples were collected at baseline for the screening of meningitis and sepsis, and for biomarker quantification. CSF was collected into two sterile screw-cap polyprolylene tubes (FL Medical, Torreglia, Italy; Lot 17F2496); the first tube was used for biochemistry and multiplex PCR testing for meningitis pathogens, while the second was used for cell count (with correction for traumatic taps using a ratio of one WBC for every 1,000 RBCs in the CSF), Gram staining, and bacterial culture (additional details are provided in the Supplementary Methods). A minimum of 3 ml of venous blood was collected: 2 ml into BD BACTEC™ Peds Plus™/F Culture Vials (Becton, Dickinson and Company, Sparks, MD, USA) for Gram staining, automated microbial growth monitoring, and subsequent antibiotic susceptibility testing; and 1 ml into VACUETTE^®^ Z Serum Clot Activator tubes (Greiner Bio-One, Kremsmünster, Austria) for biochemistry and quantification of CRP and PCT. Following these initial procedures, and provided a sufficient volume of sample remained, residual CSF and serum samples were aliquoted and stored at -80 °C, ensuring no freeze-thaw cycles.

### Biomarker quantification

Biomarker quantification was retrospectively performed at ISGlobal’s Immune Response and Biomarkers core facility (Spain) on CSF and serum samples. The quantification of target analytes was performed using a Human Magnetic Luminex^®^ Performance Assay (Catalog No. LXSAHM-02, Lot L157447; R&D Systems). Samples were processed using appropriate dilution factors with Calibrator Diluent RD6–52 as per manufacturer’s instructions. Final detection was executed on the Luminex^®^ 100/200™ platform (Bio-Techne R&D Systems, MIN, USA).

A total of 21 host-response biomarkers related to immune activation, endothelial dysfunction, and neuronal injury, selected based on their strong prognostic performance for mortality in older children and adults, were measured using custom multiplex panels. We measured the immune and endothelial activation markers CRP, procalcitonin (PCT), angiopoietin-1(Angpt-1), Angpt-2, interferon-gamma inducible protein 10 (IP-10), interleukin (IL)-6, IL-8, IL-10, IL-12p70, IL-15, tumor necrosis factor receptor superfamily member 1A TNFRSF1A (TNFRI), triggering receptor expressed on myeloid cells-1 (TREM-1), Chitinase 3-like 1 (CHI3L1), tumor necrosis factor-related apoptosis inducing ligand (TRAIL), unique long-16 binding protein (ULBP**-**2/5/6), urokinase plasminogen activator receptor (uPAR), vascular endothelial growth factor receptor-1 (VEGFR1/Flt-1), and neutrophil gelatinase-associated lipocalin (NGAL). We also included as markers of neuronal injury the neuron-specific enolase 2 (NSE), S100 calcium-binding protein B (S100B), and microtubule-associated protein tau (tau).

Samples were analyzed according to the manufacturer’s protocols, with laboratory personnel blinded to clinical data, but without technical replicates due to insufficient volume. For each analyte, low and upper limits of quantification (LLOQ and ULOQ) were determined using the standard curve in the xPONENT software 3.1. Values outside the quantification range but yielding an extrapolated concentration by the xPONENT were retained. Measurements falling outside the LOQ and not extrapolated were imputed assigning one-third of the LLOQ when below, and 3-fold the ULOQ when above. Biomarker concentrations are reported in pg/mL.

### Study endpoints

The primary outcome was in-hospital death, revised from the original composite endpoint of mortality and critical-care need because systematic timing data for individual interventions were not available. Secondary and exploratory outcomes included meningitis and sepsis, classified as clinical (ICD-10) or laboratory-confirmed. Probable or laboratory-confirmed meningitis was defined as CSF pleocytosis ≥30 white blood cells (WBC)/mm3 and/or positive culture or PCR; while microbiologically confirmed bacterial meningitis was defined as identification of a bacterial pathogen in CSF by culture or PCR. Cases considered to represent culture contaminants by local and coordinating study team were excluded from the definition of laboratory-confirmed meningitis in all analyses. Laboratory-confirmed sepsis was defined by a positive blood culture. Perinatal asphyxia was defined as APGAR ≤5 at 5 minutes; preterm birth as ≤36 weeks’ gestation, and very low birth weight as ≤1,500 grams. The APGAR score used was the earliest available. Investigators assessing clinical outcomes were blinded to the biomarkers results.

### Statistical methods

Biomarker concentrations and prognostic performance were analyzed separately in serum and CSF samples. Participants with at least one analyte measured at baseline in serum and/or CSF were included in the respective sample sets. Highly hemolytic CSF samples with red blood cell (RBC) counts ≥100,000 cells/µL were excluded from biomarker analyses.

Categorical variables are presented in absolute and relative frequencies, while continuous variables are presented in medians and interquartile ranges (IQR). Our primary hypothesis concerned differences in biomarker concentrations between groups according to in-hospital death. Median levels of all biomarkers were compared, and false discovery rate correction was applied across this full set, providing multiplicity control for the primary analysis. Markers with p ≤ 0.05 were then carried forward to multivariable models. Biomarker concentrations were log_2_-transformed prior to analysis. Associations with in-hospital death were estimated using Firth’s penalized likelihood logistic regression, selected to reduce small-sample bias and to provide finite estimates in the presence of sparse data. Odds ratios are presented per doubling of biomarker concentration, with 95% confidence intervals (Wald). Models were fitted unadjusted and with progressive adjustment for sex, age, very low birth weight, and birth asphyxia. As these markers were selected using the same data, p values from the multivariable models are not corrected for multiple testing, these analyses are secondary and exploratory. Discrimination of individual biomarkers was assessed by the area under the receiver operating characteristic curve (AUROC) with 95% confidence intervals. To evaluate whether biomarkers added information beyond routinely available clinical variables, we compared AUROC containing variables age, sex, birth weight, and asphyxia with the same model including each biomarker in turn. Both models were fitted in the identical complete-case sample. The contribution of each biomarker (comparison between AUROC with and without the marker) was compared using p-values calculated with likelihood ratio test. Apparent AUROCs and the difference between models were corrected for optimism using the bootstrap with 1,000 replications in which the full modelling procedure was repeated in each resample and evaluated in the original data. Spearman’s rank correlation coefficients (ρ) were calculated to assess the correlation between paired serum and CSF biomarker concentrations. No imputation was performed for entirely missing samples. All analyses were performed using R software, version 4.4.1, and Stata 16 (StataCorp, College Station, TX) under a two-tailed significance levels of 0.05.

## Results

### Study population

Of the 211 participants enrolled at the Moroccan site, 179 participants with CSF and/or serum samples available for biomarkers quantification were initially included in the biomarker population. Among them, 16 participants (9%) met the primary severity endpoint of in-hospital death. Thirteen CSF samples were subsequently excluded due to RBC ≥100,000 cells/µl (eight participants retained serum samples; five had no remaining sample); thus, 174 participants contributed to the final biomarker analyses, including 14 deaths (8%). Overall, 136 participants had available serum samples (serum sample set), 148 had available CSF samples (CSF sample set) and 110 had paired CSF and serum samples, with 11, 12, and 9 participants, respectively, meeting the primary endpoint (sample sets are overlapping) (**Supplementary Figure S1**). Demographic, clinical, and laboratory characteristics are presented in **Table 1**; **Supplementary Tables S1**, **S2**. The median age at recruitment was 2.8 days (IQR 1.6-10.6), and 38% were female. The most common clinical diagnosis was sepsis (75%), followed by meningitis (25%) and asphyxia (25%). All meningitis cases fulfilled the definition of probable or laboratory confirmed by CSF WBC count (26), CSF culture (7), and/or PCR (16). Among participants with clinical sepsis, only 11% were laboratory-confirmed by blood culture. Patients who died were older, and had lower birth weight and gestational age; whereas no differences were observed for sex, antibiotic treatment before enrolment, or clinical diagnoses.

**Table 1:** Characteristics of study population according to primary outcome (in-hospital death).

Characteristics N (%) or median [IQR]^a^	Total (n= 179)	Alive (n= 163)	Death (n= 16)	p-value ^b^
Baseline Demographics and Risk Factors				
Gestational age, weeks	39 [37, 40]	39 [37, 40]	36.5 [30, 40]	**0.049**
Age, days	2.8 [1.6, 10.6]	2.6 [1.5, 9.2]	6.6 [3.2, 13.3]	**0.026**
Weight at birth, grams	3000 [2400, 3600]	3150 [2600, 3605]	1710 [1371.2, 2420]	**<0.001**
Very Low Birth Weight (≤1,500 grams)	11 (6.1)	6 (3.7)	5 (31.2)	**0.001**
Sex, Female	68 (38.0)	61 (37.4)	7 (43.8)	0.603
Antibiotic treatment before enrolment	169 (94.4)	153 (93.9)	16 (100)	0.603
Days of antibiotic treatment before enrolment	2 [1, 2]	2 [1, 2]	2 [1, 2.2]	0.450
Clinical Diagnoses				
Meningitis	45 (25.1)	40 (24.5)	5 (31.2)	0.553
Sepsis	135 (75.4)	124 (76.1)	11 (68.8)	0.546
Perinatal asphyxia^c^	35 (25.2)	30 (24)	5 (35.7)	0.342
Microbiology results				
Probable or laboratory confirmed-meningitis^d^	45 (25.1)	40 (24.5)	5 (31.2)	0.553
CSF WBC count ≥30 WBC/mm3	26 (14.5)	22 (13.5)	4 (25)	0.257
CSF culture positive^e^	7 (3.9)	7 (4.3)	0 (0)	1.000
CSF PCR positive^e^	16 (8.9)	15 (9.2)	1 (6.2)	1.000
Microbiologically confirmed bacterial meningitis^f^	17 (9.5)	17 (10.4)	0 (0)	1.000
Blood culture positive for sepsis^e^	15 (8.4)	12 (7.4)	3 (18.8)	0.137
Outcome details				
Days from enrolment to death	–	–	5.1 [2.7-15.5]	–

^a^Data presented as frequency (percent) or median [interquartile range] as appropriate.

^b^p-values computed using the χ² or Fisher’s exact test for categorical variables, and the Mann-Whitney U test for continuous variables. p-values <0.05 are shown in bold.

^c^Missing data for perinatal asphyxia: (n=40).

^d^Probable or laboratory confirmed meningitis defined as WBC count ≥30 WBC/mm3 and/or positive culture or PCR.

^e^Pathogens identified in CSF and serum are described in **Supplementary Table S2**.

^f^Microbiologically confirmed bacterial meningitis defined as identification of a bacterial pathogen in CSF by culture or PCR.

### Serum biomarkers as predictors of in-hospital death

Participants who died during hospitalization showed higher baseline serum concentrations of Angpt-2, IL-8, uPAR, IL-10, IL-15 and lower concentrations of TRAIL (**Supplementary Table S3**). We did not find differences across severity groups in levels of CRP and PCT (both included in the enrolment criteria), nor in IL-6, IL-12p70, ULBP-2/5/6, CHI3L1, IP-10, TNFR1, TREM-1, NGAL, Tau, S100B, NSE, Angpt-1, or VEGFR1/Flt-1. After adjustment for multiple testing, evidence remained strong only for TRAIL, Angpt-2, IL-8, and uPAR (**Figure 1**; **Supplementary Table S3**). Baseline serum biomarker concentrations did not differ between patients with and without laboratory-confirmed sepsis (**Supplementary Figure S2**).

**Figure 1:**
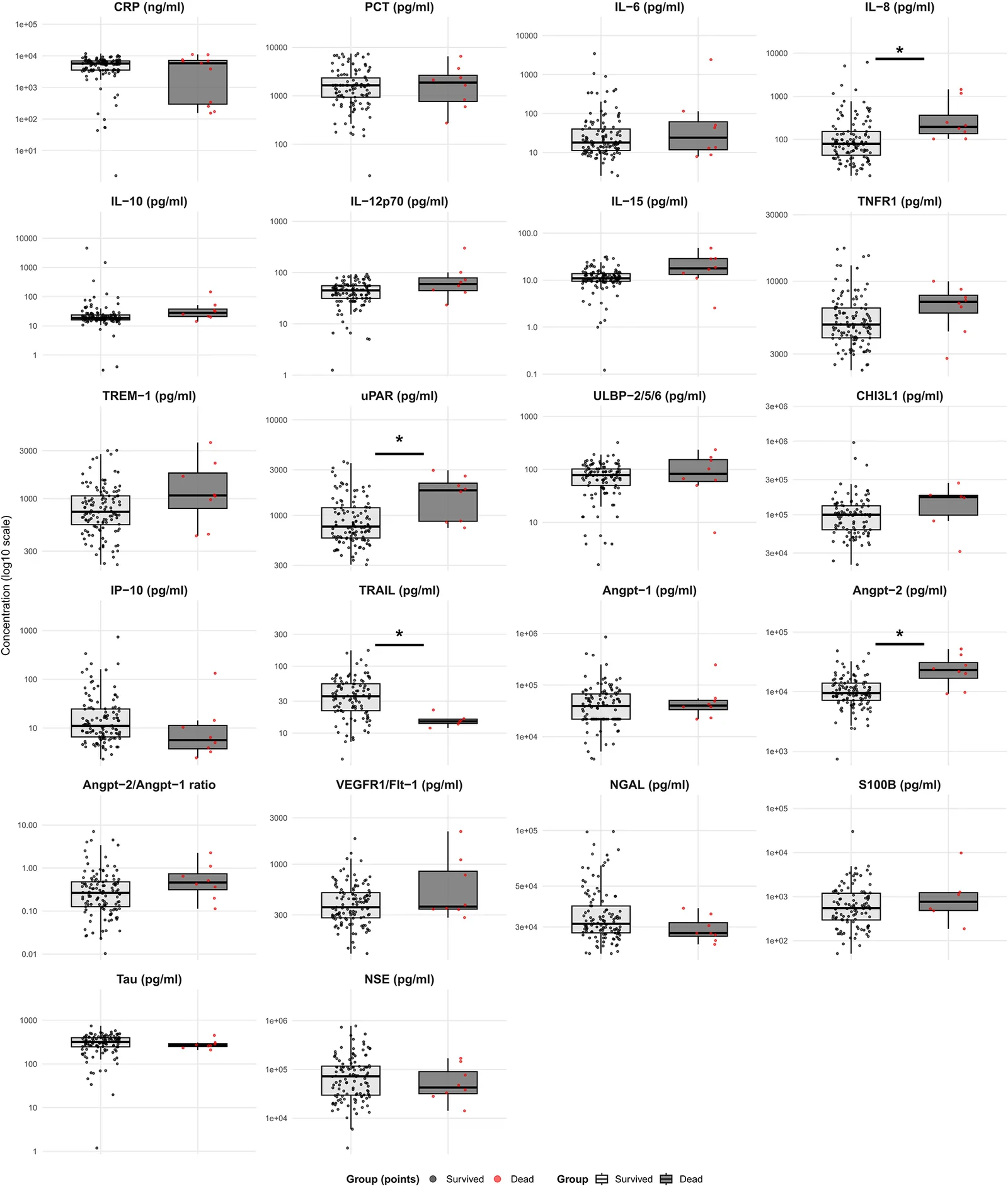
Distribution of serum concentrations of biomarkers according to in-hospital death. Boxplots showing the distribution of serum concentrations of immune and endothelial biomarkers of interest with in-hospital death: (i) survived in clear grey, and (ii) dead in dark grey. Boxes represent the interquartile range (IQR), with horizontal lines indicating the median values. Each dot corresponds to an individual patient. Adjusted p-values for multiple testing were calculated using the false discovery rate (FDR) correction across the full set of biomarkers. Statistically significant differences are indicated with asterisks: *p<0.05, **p<0.01, ***p<0.001. n=125 survived; n=11 died. Missing data for biomarkers assessment: CRP (n=0); Angpt-2, IL-8, uPAR, IL-15, IL-10, IL-12p70, IP-10, TNFR1, TREM-1, NGAL, VEGFR1, ULBP-2/5/6, Angpt-1, PCT, IL-6, Tau, NSE (n=8 each: 5 survived and 3 died); TRAIL, CHI3L1, S100B (n = 24 each: 19 survived and 5 died).

Next, we explored association of biomarker levels with increased odds of in-hospital mortality in those showing differences in median levels between survivors and non-survivors in the adjusted analyses. We observed increased odds of in-hospital death for every two-fold increase in serum levels of IL-8, uPAR, and Angpt-2, and a decrease of TRAIL, in both unadjusted models and models adjusted for sex and age (**Table 2**). The association of uPAR was attenuated after additional adjustment for very low birth weight and asphyxia, while the association for IL-8 was also attenuated after additional adjustment for asphyxia. Only a two-fold decrease in serum TRAIL concentration remained associated with higher odds of death in the fully adjusted model including sex, age, very low birth weight, and asphyxia (**Table 2**).

**Table 2:** Associations between selected serum and CSF biomarker concentrations and primary endpoint (in-hospital death) in the serum and CSF sample sets.

	Crude ^a^		Adj. by sex and age ^b^		Adj. by sex, age, and asphyxia ^c^		Adj. by sex, age, and very low birth weight ^d^		Adj. by sex, age, asphyxia, and very low birth weight ^e^	
	OR (95% CI)	p-value	aOR1 (95% CI)	p-value	aOR2 (95% CI)	p-value	aOR3 (95% CI)	p-value	aOR4 (95% CI)	p-value
SERUM										
Angpt-2	4.70 (1.89-11.73)	<0.001	4.87 (1.84–12.88)	0.001	4.34 (1.43–13.14)	0.009	3.30 (1.15–9.44)	0.026	2.54 (0.72–8.97)	0.148
IL-8	1.50 (1.07-2.11)	0.018	1.51 (1.07–2.14)	0.019	1.46 (1.00–2.14)	0.051	1.53 (1.03–2.28)	0.037	1.39 (0.89–2.20)	0.151
TRAIL	0.34 (0.14-0.80)	0.014	0.35 (0.14–0.85)	0.020	0.30 (0.11–0.85)	0.023	0.32 (0.11–0.91)	0.033	0.29 (0.09–0.94)	0.038
uPAR	3.19 (1.34-7.55)	0.009	3.06 (1.20–7.78)	0.019	2.47 (0.91–6.69)	0.076	1.97 (0.76–5.10)	0.165	1.66 (0.62–4.43)	0.316
CSF										
PCT	4.90 (2.13–11.31)	<0.001	6.10 (2.49–14.96)	<0.001	4.60 (1.72-12.27)	0.002	5.76 (2.27-14.59)	<0.001	4.07 (1.49-11.12)	0.006

^a^ORs, 95% CI and p-values were obtained from univariable Firth’s penalized likelihood logistic regression models. n/N (events/total): 8/128 for serum Angpt-2, IL-8 and uPAR, 6/112 for serum TRAIL, and 12/145 for CSF PCT.

^b^aOR1: ORs, 95% CI and p-values were obtained from multivariable Firth’s penalized likelihood logistic regression models adjusted for sex and age. n/N (events/total): 8/128 for serum Angpt-2, IL-8 and uPAR, 6/112 for serum TRAIL, and 12/145 for CSF PCT.

^c^aOR2: ORs, 95% CI and p-values were obtained from multivariable Firth’s penalized likelihood logistic regression models adjusted for sex, age and asphyxia. n/N (events/total): 7/102 for serum Angpt-2, IL-8 and uPAR, 6/102 for serum TRAIL and 12/121 for CSF PCT.

^d^aOR3: ORs, 95% CI and p-values were obtained from multivariable Firth’s penalized likelihood logistic regression models adjusted for sex, age and very low birth weight. n n/N (events/total): 8/128 for serum Angpt-2, IL-8 and uPAR, 6/112 for serum TRAIL, and 12/145 for CSF PCT.

^e^aOR4: ORs, 95% CI and p-values were obtained from multivariable Firth’s penalized likelihood logistic regression models adjusted for sex, age, asphyxia, and very low birth weight. n/N (events/total): 7/102 for serum Angpt-2, IL-8 and uPAR, 6/102 for serum TRAIL and 12/121 for CSF PCT.

Biomarker concentrations were log_2_-transformed prior to analysis. OR are presented per doubling of biomarker concentration, with 95% confidence intervals (Wald). P-values from multivariable models were not adjusted for multiple testing because biomarkers were selected for inclusion based on the same dataset.

The serum biomarker with the best discrimination capacity for in-hospital death was TRAIL (AUROC: 0.863; 95% CI: 0.784-0.934), followed by Angpt-2 (AUROC 0.844; 95% CI: 0.670-0.974), IL-8 (AUROC 0.797; 95% CI: 0.699-0.893), and uPAR (AUROC 0.785; 95% CI 0.627-0.927)(**Figure 2A**). As exploratory analyses, we assessed the clinical value of serum TRAIL levels for prognostic assessment by adding it to a clinical model including age, sex, birth weight, and asphyxia. TRAIL significantly increased the AUROC from 0.776 (95% CI 0.528-1.00; corrected AUROC 0.716) in the clinical model alone to 0.902 (95% CI 0.798-1.00; corrected AUROC 0.849) (ΔAUROC=0.126; 95% CI 0.031-0.271; corrected difference 0.133; p=0.018) (**Supplementary Table S4**).

**Figure 2:**
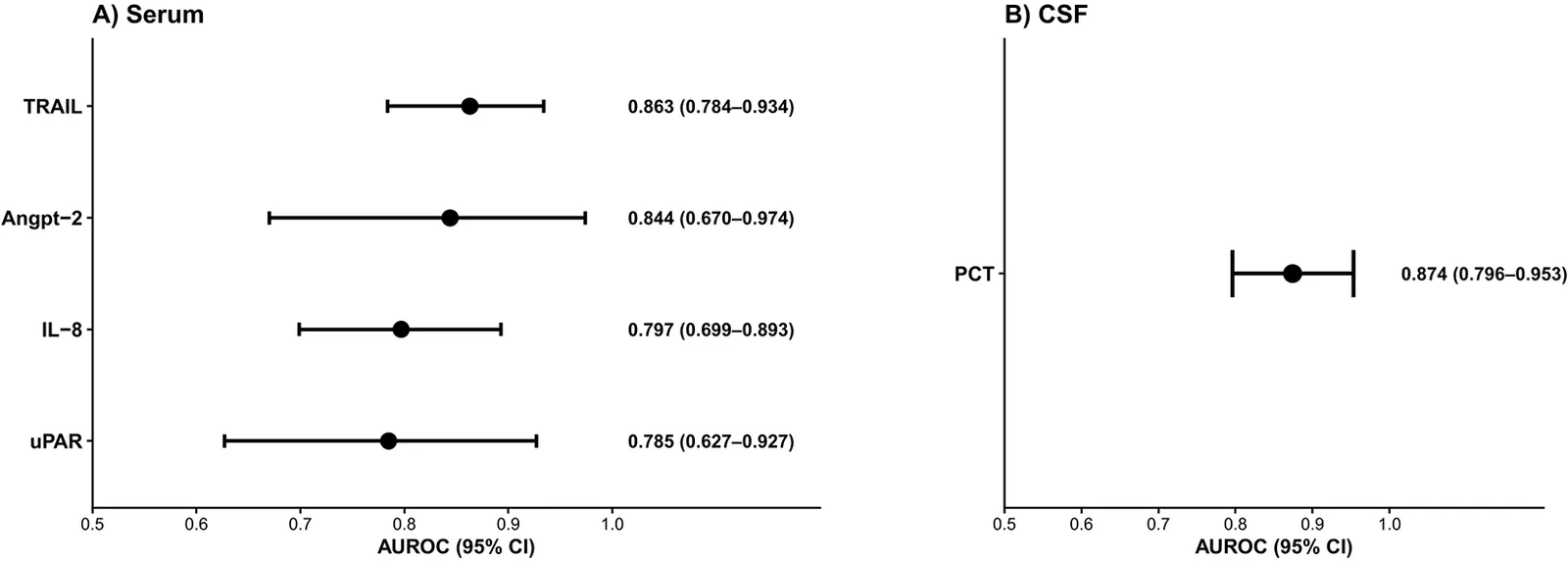
AUROC of selected serum and CSF biomarkers for predicting in-hospital death. **(A)** Forest plot showing AUROC for in-hospital death of selected serum biomarkers: uPAR, IL-8, TRAIL, and Angpt-2. Dots show optimism-corrected AUROC estimates using 1,000 bootstrap replications, with lines indicating 95% CI. n/N (events/total): 8/128 for serum Angpt-2, IL-8 and uPAR, 6/112 for serum TRAIL. **(B)** Forest plot showing AUROC for in-hospital death of selected CSF biomarker PCT. Dots show optimism-corrected AUROC estimates using 1,000 bootstrap replications, with lines indicating 95% CI. CSF sample n/N (events/total): 12/145 for CSF PCT.

### CSF biomarkers as predictors of in-hospital death

Participants who died during hospitalization showed higher baseline CSF levels of PCT, TREM-1, and TNFR1 (**Supplementary Table S5**). After adjustment for multiple testing, only PCT remained associated with the primary endpoint (**Figure 3**).

**Figure 3:**
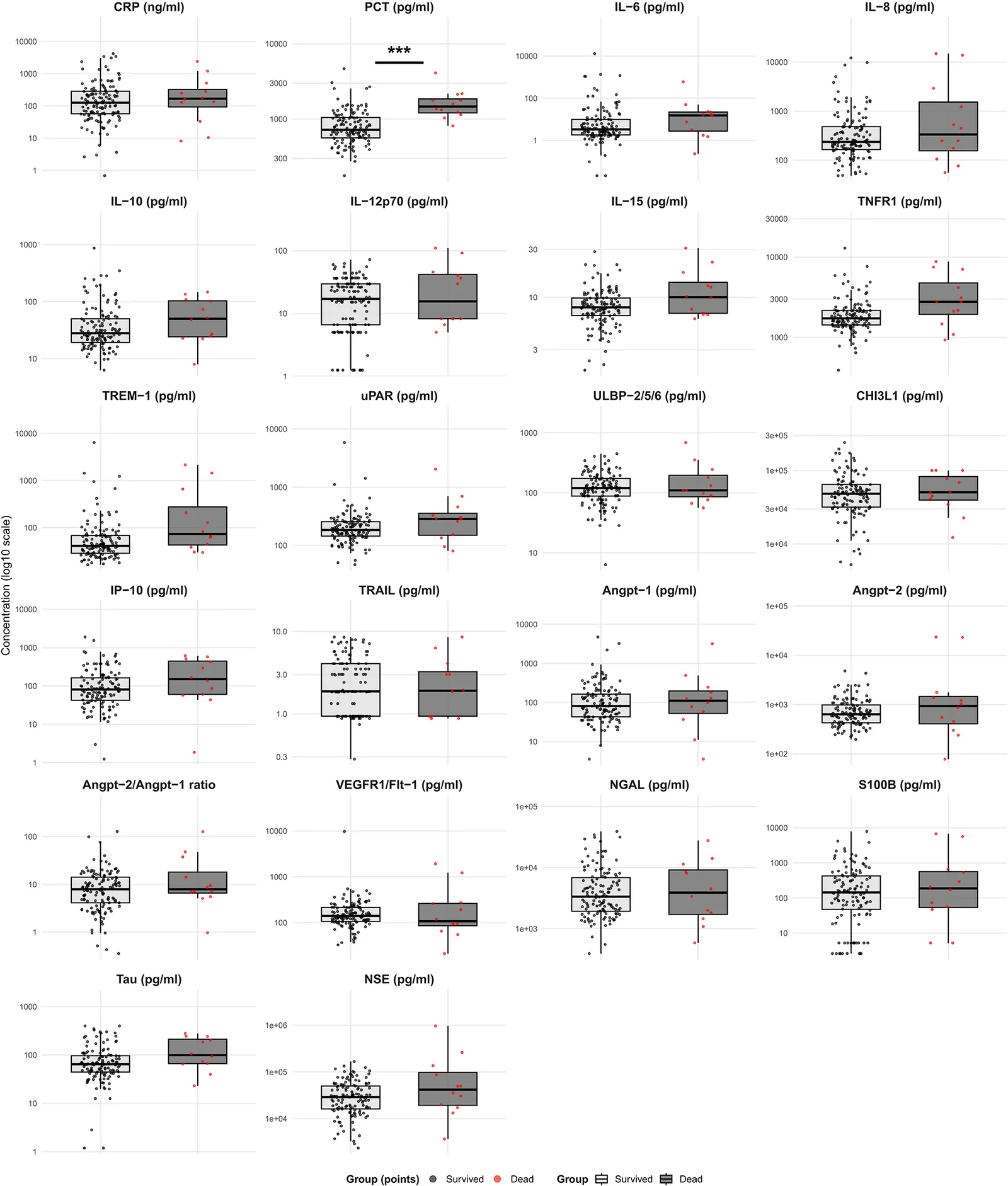
Distribution of CSF concentrations of biomarkers according to in-hospital death. Boxplots showing the distribution of CSF concentrations of immune and endothelial activation biomarkers of interest with in-hospital death: (i) survived in clear grey, and (ii) dead in dark grey. Boxes represent the interquartile range (IQR), with horizontal lines indicating the median values. Each dot corresponds to an individual patient. Adjusted p-values for multiple testing were calculated using the false discovery rate (FDR) correction across the full set of biomarkers. Statistically significant differences are indicated with asterisks: *p<0.05, **p<0.01, ***p<0.001. n=136 survived; n=12 died. Missing data for biomarkers assessment: CRP (n=0); PCT, TREM-1, TNFR1, Tau, IL-15, IL-6, NSE, uPAR, IL-12p70, IL-10, IL-8, IP-10, Angpt-2, Angpt-1, ULBP-2/5/6, NGAL, VEGFR1 (n=3 each: 3 survived and 0 died); TRAIL, CHI3L1, S100B (n = 12 each: 12 survived and 0 died).

We found strong evidence of increased odds of in-hospital death for every two-fold increase in CSF levels of PCT, which remained after adjusting for age, sex, very low birth weight, and perinatal asphyxia (**Table 2**).

We also explored the discrimination capacity of CSF PCT for in-hospital mortality, which showed good performance with an AUROC of 0.874 (95% CI: 0.796-0.953) (**Figure 2B**). As exploratory analyses, when added to a clinical model including age, sex, birth weight, and asphyxia, PCT significantly increased the AUROC from 0.827 (95% CI 0.682-0.971; corrected AUROC 0.778) to 0.892 (95% CI 0.791-0.993; corrected AUROC 0.850) (ΔAUROC=0.065; 95% CI 0.012-0.176; corrected difference 0.072; p=0.004) (**Supplementary Table S4**).

### Paired serum and CSF analyses

Spearman correlation analysis between serum and CSF biomarker concentrations is shown in **Supplementary Figure S3**. Moderate positive correlations were observed for ULBP-2/5/6 (ρ = 0.50), TNFR1 (ρ = 0.49), TREM-1 (ρ = 0.46) and NGAL (ρ = 0.46). Weak correlations (ρ <0.40) were observed for PCT, IL-6, IL-8, IL-10, IL-12p70, IL-15, uPAR, CHI3L1, IP-10, Angpt-2, NGAL, and VEGFR1, while no significant correlations were observed for CRP, Angpt-1, NSE, Tau, S100B, and TRAIL. Restricting analyses to participants with paired serum and CSF samples did not substantially alter the findings. In serum, TRAIL remained the biomarker with the highest discriminative performance for predicting death (AUROC 0.85; 95% CI: 0-75-0.95), whereas in CSF, PCT remained the best-performing biomarker (AUROC: 0.85; 95% CI: 0.73-0.98) (**Supplementary Table S6**). Combining serum TRAIL with CSF PCT did not significantly improve discrimination of in-hospital mortality compared with serum TRAIL alone (AUROC 0.85; 95% CI 0.75-0.95; p=0.896).

### Serum and CSF biomarkers associated with meningitis

Serum baseline biomarker concentrations did not differ between patients with and without probable or laboratory-confirmed meningitis (**Supplementary Table S7**). In contrast, CSF levels of IL-6, IL-12p70, TNFR1, TREM-1, Angpt-1, NGAL, and NSE were higher among patients with probable or laboratory-confirmed meningitis (**Supplementary Table S8**). A two-fold increase in each of these biomarkers was associated with higher odds of probable or laboratory-confirmed meningitis, and these associations remained after adjustment for age, sex, and very low birth weight (**Supplementary Table S9**). In analyses restricted to microbiologically confirmed bacterial meningitis, only IL-6 levels were higher among participants with meningitis (**Supplementary Table S10**). A two-fold increase in CSF IL-6 was associated with higher odds of microbiologically confirmed bacterial meningitis in both unadjusted and adjusted models (adjusted for age, sex, and very low birth weight) (**Supplementary Table S11**). On the other hand, we observed higher CSF levels of PCT and uPAR in participants that died among those with probable or laboratory-confirmed meningitis (**Supplementary Table S12**).

## Discussion

In this secondary analysis of a prospective cohort of 179 hospitalized neonates and young infants (≤2 months of age) with suspected SBIs, baseline serum levels of TRAIL, IL-8, uPAR, and Angpt-2 differed according to progression to in-hospital death, with TRAIL showing the highest prognostic performance. In CSF, PCT was the strongest predictor of severe progression. Our results align with extensive evidence from older pediatric and adult populations showing that levels of markers of immune activation and inflammation are strongly associated with adverse outcomes across a wide range of infectious diseases. Importantly, we extended these observations to a population with high disease severity and limited prognostic biomarker data, showing an exploratory potential to improve discrimination of severity beyond clinical models based on established maternal and neonatal risk factors for morbidity and death (44). In addition, the simultaneous evaluation of serum and CSF biomarkers provides novel insight into compartment-specific host responses, an area with limited prior evidence in early infancy. TRAIL, a member of the TNF superfamily and key immune modulator that induces apoptosis in activated immune and infected cells (45–47), emerged as the individual best prognostic biomarker in serum. TRAIL is typically upregulated during viral infections (48) and has been validated for the differential diagnosis of viral and bacterial infections (49). Lower circulating levels have been also associated with septic shock, organ dysfunction, lower lymphocyte and monocyte counts, and mortality in adult and older pediatric sepsis cohorts (50, 51). Decreased TRAIL levels have also been linked to longer hospital and ICU stay, prolonged oxygen therapy, and the need for mechanical ventilation in children with respiratory tract infections (52), and in adults with COVID-19 (53). Consistent with these observations, our findings support low TRAIL serum levels as a marker of severe disease progression and mortality in neonates and young infants hospitalized with sepsis and meningitis.

Other immune response biomarkers, including uPAR, IL-8, Angpt-2, and TREM-1 have also demonstrated robust prognostic value for disease severity in older children with sepsis, pneumonia, malaria, and undifferentiated febrile illness in LMICs (16, 17, 21, 22, 54–56). In particular, TREM-1 has consistently ranked among the top predictors of disease severity in several prospective comparative biomarker studies in older febrile hospitalized and non-hospitalized children (17, 22, 55, 56), outperforming established clinical scores (SOFA, qSOFA, LODS) and conventional inflammatory markers (CRP, PCT). Interestingly, in our cohort of hospitalized neonates and young infant, most clinically diagnosed with bacterial sepsis, serum levels of TRAIL outperformed those of TREM-1. These findings may partly relate to the biological matrix used, as our analyses were conducted in serum, whereas previous TREM-1 studies have predominantly used EDTA plasma. Clotting can sequester certain analytes and reduce their measured concentrations, potentially leading to poorer prognostic performance in serum compared with plasma. TRAIL may be less affected by clotting compared to other markers, which could partly explain its stronger performance in our study. Differences in study populations may also contribute. Our cohort comprised hospitalized neonates and young infants with relatively advanced, predominantly bacterial disease, whereas biomarkers assessed earlier or in populations with older age, milder illness, or a higher proportion of viral infections may yield different results. Further studies directly comparing serum and plasma samples across diverse clinical contexts are needed to validate these findings.

Evidence on prognostic markers in neonates and young infants remains limited and largely derived from small studies. In line with our findings, elevated sTREM-1 levels were associated with septic shock or death in a cohort of 71 neonates with late-onset sepsis (41); while IL-8 was strongly predictive of mortality in a prospective cohort of 59 neonates (38). However, unlike our results, IL-6 emerged as the best performing mortality prognostic marker in two large neonatal studies in Benin and the Netherlands, where biomarkers were measured in cord blood or in preterm populations (34, 40). IL-6 and IL-10 also showed a good predictive accuracy for the development of disseminated intravascular coagulation in neonatal sepsis in preterm infants (37). These discrepancies might reflect differences in study design and context, including the biological matrix, population characteristics, timing of sampling, prior antibiotic exposure, and underlying etiologies. Importantly, the intended use of each biomarker along the patient pathway should be considered: some markers may perform better early in the disease course (CRP, IL-6), whereas others may better reflect later disease severity (TREM-1, TRAIL). Together, these findings underscore the complexity of neonatal immune responses and highlight the need for larger context-specific biomarker validation studies.

Of note, we evaluated immune activation and neuronal injury biomarkers in CSF for prediction of adverse outcomes and diagnosis of meningitis. CSF PCT showed the strongest prognostic performance for in-hospital death. TREM-1, IL-6, IL-12p70, TNFR1, Angpt-1, NGAL, and NSE demonstrated good discrimination for probable or laboratory-confirmed meningitis, while IL-6 was associated with microbiologically confirmed bacterial meningitis. Previous studies have reported excellent diagnostic accuracy of CSF PCT and IL-6 for distinguishing bacterial from nonbacterial meningitis and from non-central nervous system (CNS) infections (57–60). Notably, blood biomarkers have shown limited discrimination in pediatric bacterial meningitis (57), supporting the added value of CSF-based assessment observed in our study. Elevated CSF NSE has also been linked to adverse outcomes in neonatal sepsis-associated encephalopathy, reflecting hypoxia-induced neuronal damage (61); as well as mortality in pediatric tuberculous meningitis (62). Overall, the prognostic role of CSF biomarkers in neonates and young infants undergoing lumbar puncture remains largely unexplored, and our findings contribute to initially addressing this gap.

Our study has some limitations. First, elevated serum CRP and PCT levels were part of the inclusion criteria, precluding evaluation of their independent prognostic value and comparison with the remaining markers in a population of broader severity levels. Second, clinical scores were not systematically recorded, preventing comparison with established risk stratification tools. We assessed the incremental value of biomarkers beyond clinical variables; however, the small number of deaths limits the precision of these exploratory analyses and warrants cautious interpretation. Third, biomarkers were assessed in serum, as many collected plasma samples exhibited hemolysis and were unsuitable for analysis, precluding comparisons between matrices. Fourth, serum and CSF biomarkers were analyzed in partially overlapping populations with modest differences in diagnostic distribution that may have influenced performance estimates. Fifth, timing of symptom onset and admission to sample collection was unavailable, and biomarkers were measured at a single timepoint, limiting assessment of biomarker kinetics. Sixth, most participants had received antibiotics prior to sampling, which may have affected biomarker concentrations, although this reflects routine clinical practice. Finally, the predominance of sepsis diagnoses limited power for diagnosis-specific prognostic analyses; while the small number of confirmed viral infections precluded meaningful subgroup analyses for differential diagnosis of viral versus bacterial infections.

In this secondary analysis of a prospective cohort of hospitalized neonates and young infants with suspected serious bacterial infections, lower TRAIL, and higher Angpt-2, IL-8, and uPAR concentrations in serum, were associated with progression to in-hospital death, with TRAIL showing the highest discriminatory capacity. Assessment of CSF biomarkers extended these findings, with CSF PCT showing the strongest association with death, while IL-6, IL-12p70, TNFR1, TREM-1, Angpt-1, NGAL, and NSE were associated with meningitis, warranting further investigation. Our findings provide exploratory evidence of associations between immune activation markers and mortality in sick neonates and young infants. Validation in larger, longitudinal, multicenter cohorts using multiple biological matrices is needed to assess reproducibility, characterize biomarker kinetics, and define clinically relevant thresholds. Further studies are also needed to determine whether these biomarkers could ultimately contribute to risk stratification and timely identification of high-risk infants, guiding escalation of care, and improving outcomes, particularly in LMICs where preventable neonatal mortality remains highest.

## Data Availability

The original contributions presented in the study are included in the article/**Supplementary Material**. Further inquiries can be directed to the corresponding authors.
