# Supplementary material for: Serum and cerebrospinal fluid biomarkers for risk stratification of hospitalized neonates and young infants with suspected serious bacterial infections: a secondary analysis of a prospective cohort study
Source: Front Immunol. 2026 Oct 16;17:1873954. doi: 10.3389/fimmu.2026.1873954 (PMC13719337; doi:10.3389/fimmu.2026.1873954)
Supplement: Supplementary file 1 [file DataSheet1.pdf]

Supplementary appendix to Serum and cerebrospinal fluid biomarkers for risk stratification of hospitalized neonates and young infants with suspected serious bacterial infections: a secondary analysis of a prospective cohort study

Table of contents

Supplementary Methods 1. Meningitis PCR panel, culture procedures, and contaminant-adjudication criteria....2

Figure S1. Study flowchart....3

Table S1. Characteristics of serum and CSF study populations according to primary outcome (in-hospital death)....4

Table S2. Microbiology results of study population....6

Table S3. Distribution of serum concentrations of biomarkers according to in-hospital death....7

Figure S2. Distribution of serum concentrations of biomarkers according to laboratory-confirmed bacterial sepsis....8

Table S4. Incremental discrimination of selected biomarkers beyond clinical variables for prediction of in-hospital death....9

Table S5. Distribution of CSF concentration of biomarkers according to in-hospital death....10

Figure S3. Correlation of biomarker concentrations between paired serum and CSF samples....11

Table S6. AUROC of selected serum and CSF biomarkers for predicting in-hospital death in paired samples....12

Table S7. Distribution of serum concentrations of biomarkers according to the diagnosis of probable or laboratory-confirmed meningitis....13

Table S8. Distribution of CSF concentrations of biomarkers according to the diagnosis of probable or laboratory-confirmed meningitis....14

Table S9. Associations between CSF biomarker concentrations and probable or laboratory-confirmed meningitis in the CSF study population....15

Table S10. Distribution of CSF concentrations of biomarkers according to the diagnosis of microbiologically confirmed bacterial meningitis....16

Table S11. Associations between CSF biomarker concentrations and microbiologically confirmed bacterial meningitis in the CSF study population....17

Table S12. Distribution of CSF biomarker concentrations according to in-hospital death in patients with probable or laboratory-confirmed meningitis....18

Supplementary Methods 1. Meningitis PCR panel, culture procedures, and contaminant-adjudication criteria

Cerebrospinal fluid (CSF) samples were investigated for bacterial and viral pathogens using molecular and microbiological methods. For molecular testing, bacterial and viral pathogens were detected using the FilmArray Meningitis/Encephalitis (ME) Panel (BioFire Diagnostics), a multiplex PCR assay targeting 14 pathogens. The panel detects seven viruses (i.e., *cytomegalovirus [CMV], enterovirus, herpes simplex virus 1 [HSV-1], herpes simplex virus 2 [HSV-2], human herpesvirus 6 [HHV-6], human parechovirus, and varicella-zoster virus [VZV]*), six bacteria (i.e., *Escherichia coli K1, Haemophilus influenzae, Listeria monocytogenes, Neisseria meningitidis, Streptococcus agalactiae, and Streptococcus pneumoniae*), and one yeast (i.e., *Cryptococcus neoformans/gattii*). Importantly, the FilmArray ME Panel targets a predefined set of 14 pathogens and does not detect all bacterial species that may cause CNS infection. Therefore, a negative FilmArray ME result was not interpreted as evidence of contamination when an organism not included among the panel targets was recovered by CSF culture. In our cohort, these organisms included *Klebsiella pneumoniae, Serratia marcescens, Staphylococcus aureus,* and coagulase-negative staphylococci.

CSF samples were cultured using standard microbiological procedures. Bacterial isolates were identified using the BD Phoenix automated identification system (BD Diagnostics), according to the manufacturer's instructions and standard laboratory procedures. The system was used for the identification of clinically relevant bacterial isolates, including *Listeria monocytogenes*, *Neisseria meningitidis*, *Streptococcus agalactiae, Streptococcus pneumoniae,* and *Haemophilus influenzae*, as well as other clinically relevant Gram-positive and Gram-negative organisms when isolated.

For contaminant adjudication, bacterial isolates recovered from CSF cultures were assessed using the available clinical, CSF, and microbiological information to distinguish clinically significant isolates from potential contaminants. The assessment considered four main elements: (1) organism identity and its likelihood of representing a true CNS pathogen versus a common skin or environmental contaminant; (2) available CSF findings, including leukocyte count and glucose and protein concentrations, as well as any clinical findings compatible with CNS infection documented at or before the time of lumbar puncture; (3) microbiological concordance, including recovery of the same organism from blood culture, repeated isolation of the same organism from an independent CSF specimen, and direct Gram staining; and (4) concordance with molecular testing when the organism was included among the FilmArray ME Panel targets. Organism identity was considered an important component of the adjudication. Organisms with a low a priori likelihood of contamination, including *Klebsiella pneumoniae, Serratia marcescens,* and *Staphylococcus aureus*, were interpreted in conjunction with the available CSF, clinical, and microbiological findings. Coagulase-negative staphylococci were considered potential contaminants when isolated from a single CSF specimen without supporting microbiological or clinical evidence. Conversely, concordant recovery of the same organism from CSF and blood was considered strong microbiological evidence supporting the clinical significance of the CSF isolate, including for coagulase-negative staphylococci. The absence of CSF pleocytosis alone was not considered sufficient to classify a culture-positive result as a contaminant; the interpretation also took into account organism identity and the available clinical and microbiological findings.

Figure S1. Study flowchart

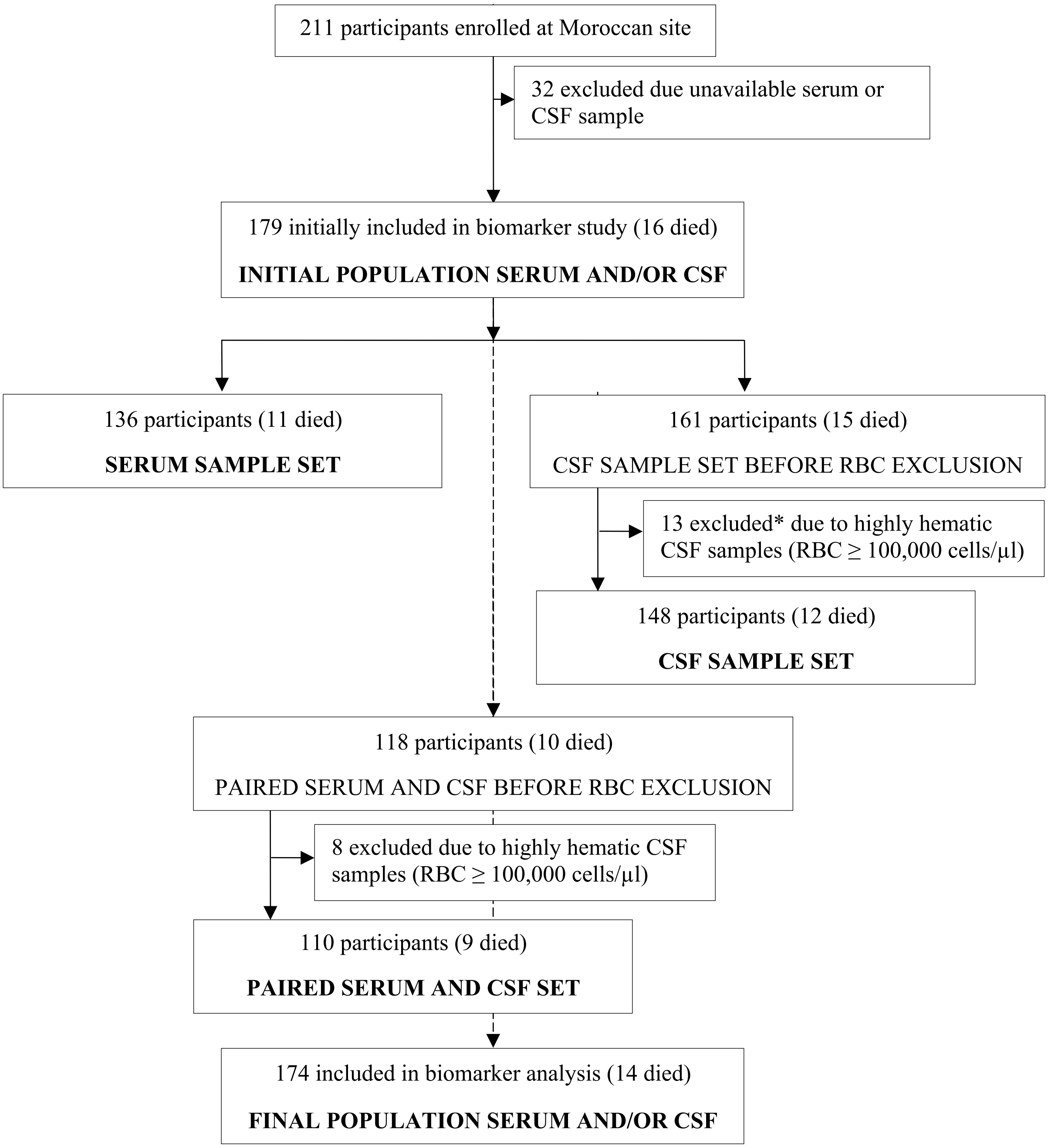

**Missing data for biomarkers assessment**			
Biomarkers	Serum sample set	CSF sample set	Paired serum and CSF
CRP	0	0	0
Angpt-2, IL-8, uPAR, IL-15, IL-10, IL-12p70, IP-10, TNFR1, TREM-1, NGAL, VEGFR1, ULBP-2/5/6, Angpt-1, PCT, IL-6, Tau, NSE	8 (3 died)	3 (0 died)	11 (3 died)
TRAIL, CHI3L1, S100B	24 (5 died)	12 (0 died)	28 (5 died)

Legend: The serum and CSF sample sets overlap; participants with paired serum and CSF samples are included in both sets. Therefore, these groups are not mutually exclusive.
*13 excluded: 8 with retained serum sample (remained in serum sample set) and 5 with no remaining sample for biomarker analysis, and therefore excluded from the final analytical population.

Table S1. Characteristics of serum and CSF study populations according to primary outcome (in-hospital death)

CHARACTERISTICS<br>N (%) or median [IQR][a]	TOTAL<br>(n=179)	Survived<br>(n=163)	Died<br>(n=16)	p-value [b]
**A. SERUM POPULATION**	**(n=136)**	**(n=125)**	**(n=11)**	
Baseline Demographics and Risk Factors				
Gestational age, weeks	39 [37, 40]	39 [38, 40]	39 [30, 40]	0.143
Age, days	2.7 [1.6-8.6]	2.6 [1.5, 6.8]	6.8 [3.7, 12.4]	**0.017**
Weight at birth, grams	3000 [2437.5–3620]	3050 [2600, 3650]	1720 [1347.5, 3050]	**0.013**
Very Low Birth Weight (≤1,500 grams)	10 (7.4)	6 (4.8)	4 (36.4)	**0.004**
Sex, Female	54 (39.7)	49 (39.2)	5 (45.5)	0.753
Antibiotic treatment before enrolment	129 (94.9)	118 (94.4)	11 (100.0)	1.000
Days of antibiotic treatment before enrolment	2 [1, 2]	2 [1, 2]	2 [1, 2]	0.566
**Clinical Diagnoses**				
Meningitis	33 (24.3)	31 (24.8)	2 (18.2)	1.000
Sepsis	104 (76.5)	95 (76.0)	9 (81.8)	1
Perinatal asphyxia[c]	24 (22.2)	20 (20.2)	4 (44.4)	0.108
**Microbiology results**				
Probable or laboratory confirmed-meningitis[d]	33 (24.3)	31 (24.8)	2 (18.2)	1.000
CSF WBC count ≥30 WBC/mm3	18 (13.2)	16 (12.8)	2 (18.2)	0.640
CSF culture positive	7 (5.1)	7 (5.6)	0 (0)	1.000
CSF PCR positive	11 (8.1)	11 (8.8)	0 (0)	0.599
Microbiologically confirmed bacterial meningitis[e]	15 (11.03)	15 (12)	0 (0)	1.000
Blood culture positive for sepsis	13 (9.6)	11 (8.8)	2 (18.2)	0.283
**Outcome details**				
Days from enrolment to death	-	-	5 [2.1-8.9]	
**B.CSF POPULATION**	**(n=148)**	**(n=136)**	**(n=12)**	
Baseline Demographics and Risk Factors				
Gestational age, weeks	39.0 [37.0-40.0]	39.0 [37.0-40.0]	33 [30.0-39.2]	**0.019**
Age, days	2.7 [1.5-11.7]	2.5 [1.4-11.1]	9.6 [5.8-16.1]	**0.005**
Weight at birth, grams	3075.0 [2400.0-3600.0]	3150.0 [2600.0-3602.5]	1625.0 [1353.8-2105.0]	**<0.001**
Very Low Birth Weight (≤1,500 grams)	9 (6.1)	5 (3.7)	4 (33.3)	**0.003**
Sex, Female	55 (37.2)	48 (35.3)	7 (58.3)	0.129
Antibiotic treatment before enrolment	139 (93.9)	127 (93.4)	12 (100.0)	1.000
Days of antibiotic treatment before enrolment	2.0 [1.0-2.0]	2.0 [1.0-2.0]	2.0 [1.0-2.5]	0.604
**Clinical Diagnoses**				
Meningitis	36 (24.3)	33 (24.3)	3 (25.0)	1.000
Sepsis	113 (76.4)	104 (76.5)	9 (75.0)	1.000
Perinatal asphyxia[c]	31 (27.2)	28 (26.9)	3 (30.0)	1.000
**Microbiology results**				
Probable or laboratory confirmed-meningitis[d]	36 (24.3)	33 (24.3)	3 (25.0)	1.000
CSF WBC count ≥30 WBC/mm3	18 (12.2)	16 (11.8)	2 (16.7)	0.641
CSF culture positive	7 (4.7)	7 (5.1)	0 (0)	1.000
CSF PCR positive	14 (9.5)	13 (9.6)	1 (8.3)	1.000
Microbiologically confirmed bacterial meningitis [e]	16 (10.8)	16 (11.8)	0 (0)	1.000

Blood culture positive for sepsis	12 (8.1)	10 (7.4)	2 (16.7)	0.252
**Outcome details**				
Days from enrolment to death	-	-	4.6 [2.1-12.7]	-

[a] Data presented as frequency (percent) or median [interquartile range] as appropriate.
[b] p-values computed using the χ^2 or Fisher's exact test for categorical variables, and the Mann-Whitney U test for continuous variables. p-values <0.05 are shown in bold.
[c] Missing data for perinatal asphyxia: serum (n=28); CSF (n=35).
[d] Probable or laboratory-confirmed meningitis defined as WBC count ≥30 WBC/mm3 and/or positive culture or PCR.
[e] Microbiologically confirmed bacterial meningitis defined as identification of a bacterial pathogen in CSF by culture or PCR
13 CSF samples with RBC >100,000 cells/µL were excluded from these analyses

Table S2. Microbiology results of study population

CHARACTERISTICS<br>N (%) or median [IQR][a]	Survived *<br>(n=163)	Died<br>(n=16)
Laboratory confirmed-meningitis[b]	40 (24.5)	5 (31.2)
CSF WBC count ≥30 WBC/mm3	22 (13.5)	4 (25)
CSF culture positive	7 (4.3)<br>*Klebsiella pneumoniae* (n=2)[c]<br>*Serratia marcesens* (n=1)<br>*Staphylococcus aureus* (n=1)<br>*Staphylococcus coagulase negative* (n=1)<br>*Staphylococcus hominis* (n=1)<br>*Staphylococcus epidermis* (n=1)<br>*Staphylococcus haemolyticus* (n=1)[c]	0 (0)
CSF PCR positive	15 (9.2)	1 (6.2)
CSF PCR positive Bacteria	10 (6.5)<br>*Haemophilus influenza* (n= 5)[d]<br>*Streptococcus agalactiae* (n=5)<br>*Streptococcus pneumonia* (n= 1)[d]	0 (0)
CSF PCR positive Virus	5 (3.07)<br>*Enterovirus* (n=2)<br>*Herpes virus human 6 (HHV6) (n=2)*<br>*Virus herpes simplex 1 (HSV-1) (n=1)*	*1 (6.2)*<br>*Cytomegalovirus (n=1)*
Blood culture positive for sepsis[e]	12 (7.4)<br>*Acinetobacter baumannii(n=1)*<br>*Coagulase-negative staphylococci (n=1)*<br>*Klebsiella pneumoniae (n=3)*[e]<br>*Staphylococcus aureus (n=1)*<br>*Staphulococcus epidermis (n=1)*<br>*Staphylococcus hominis (n=2)*<br>*Staphylococcus haemolyticus (n=3)*[e]<br>*Serratia marcesens (n=1)*	3 (18.8)<br>*Coagulase-negative staphylococci (n=1)*<br>*Klebsiella pneumoniae (n=2)*

[a] Data presented as frequency (percent) or median [interquartile range] as appropriate.
[b] Probable and laboratory confirmed meningitis defined as WBC count ≥30 WBC/mm3 and/or positive culture or PCR.
[c] Including one co- infection *Klebsiella pneumoniae* and *Staphylococcus haemolyticus*
[d] Including one co- infection *Haemophilus influenzae* and *Streptococcus pneumonia*
[e] Including one co- infection *Klebsiella pneumoniae* and *Staphylococcus haemolyticus*

Table S3. Distribution of serum concentrations of biomarkers according to in-hospital death

BIOMARKERS IN SERUM (pg/ml) Median [IQR]	Survived (n=125)	Died (n=11)	p-value [a]	adjusted p-value [b]
**Angpt-2**	9426.88 [7166.09-13896.94]	23053.55 [17552.17-31298.91]	0.001	0.026
**TRAIL**	35.36 [21.63-54.38]	15.04 [13.91-16.13]	0.003	0.032
**IL-8**	79.22 [42.94-152.28]	195.05 [137.95-477.90]	0.005	0.037
**uPAR**	762.56 [579.29-1200.20]	1827.13 [866.48-2182.55]	0.007	0.040
**IL-15**	10.98 [9.44-13.78]	17.87 [13.31-28.61]	0.016	0.072
**IL-10**	18.63 [16.15-23.50]	28.28 [20.92-38.25]	0.036	0.134
**IL-12p70**	44.83 [31.40-55.49]	60.16 [44.66-79.39]	0.059	0.186
**IP-10**	11.11 [6.58-24.65]	5.72 [3.78-11.39]	0.069	0.189
**TNFR1**	4888.60 [3920.19-6427.49]	7118.54 [5993.80-7935.55]	0.088	0.209
**Angpt-2/ Angpt-1 ratio**	0.26 [0.13-0.48]	0.47 [0.32-0.76]	0.095	0.209
**TREM-1**	741.40 [550.99-1066.42]	1076.68 [844.54-1817.67]	0.114	0.228
**NGAL**	31185.37 [27883.23-39022.89]	27741.80 [26685.42-31707.59]	0.131	0.240
**CHI3L1**	100120.00 [62259.87-131760.89]	173180.52 [104038.60-182510.90]	0.149	0.253
**VEGFR1/Flt-1**	359.31 [279.79-509.40]	364.94 [343.21-856.96]	0.209	0.329
**ULBP-2/5/6**	77.48 [49.52-101.98]	84.38 [59.36-152.21]	0.463	0.599
**Angpt-1**	39337.52 [21850.00-67297.90]	40279.47 [34178.22-50606.19]	0.590	0.683
**PCT**	1650.00 [934.43-2345.31]	1883.92 [768.11-2695.91]	0.685	0.754
**IL-6**	18.08 [11.14-40.05]	28.35 [11.91-66.12]	0.772	0.808
**CRP (ng/ml)**	5717.32 [3552.66-6893.52]	5811.11 [299.06-7205.73]	0.879	0.879
**Tau**	317.25 [245.90-396.50]	267.81 [252.01-291.72]	0.313	0.459
**S100B**	546.65 [295.52-1189.81]	816.54 [487.34-1224.83]	0.434	0.597
**NSE**	72262.62 [29622.54-116788.63]	42785.92 [31855.30-93723.19]	0.513	0.626

Missing data for biomarkers assessment: CRP (n=0); Angpt-2, IL-8, uPAR, IL-15, IL-10, IL-12p70, IP-10, TNFR1, TREM-1, NGAL, VEGFR1, ULBP-2/5/6, Angpt-1, PCT, IL-6, Tau, NSE (n=8 each: 5 survived and 3 died); TRAIL, CHI3L1, S100B (n = 24 each: 19 survived and 5 died)

[a] Unadjusted p-values calculated using the Wilcoxon rank-sum test.

[b] Adjusted p-values for multiple testing using the false discovery rate (FDR) correction across the full set of biomarkers.

Figure S2. Distribution of serum concentrations of biomarkers according to laboratory-confirmed bacterial sepsis

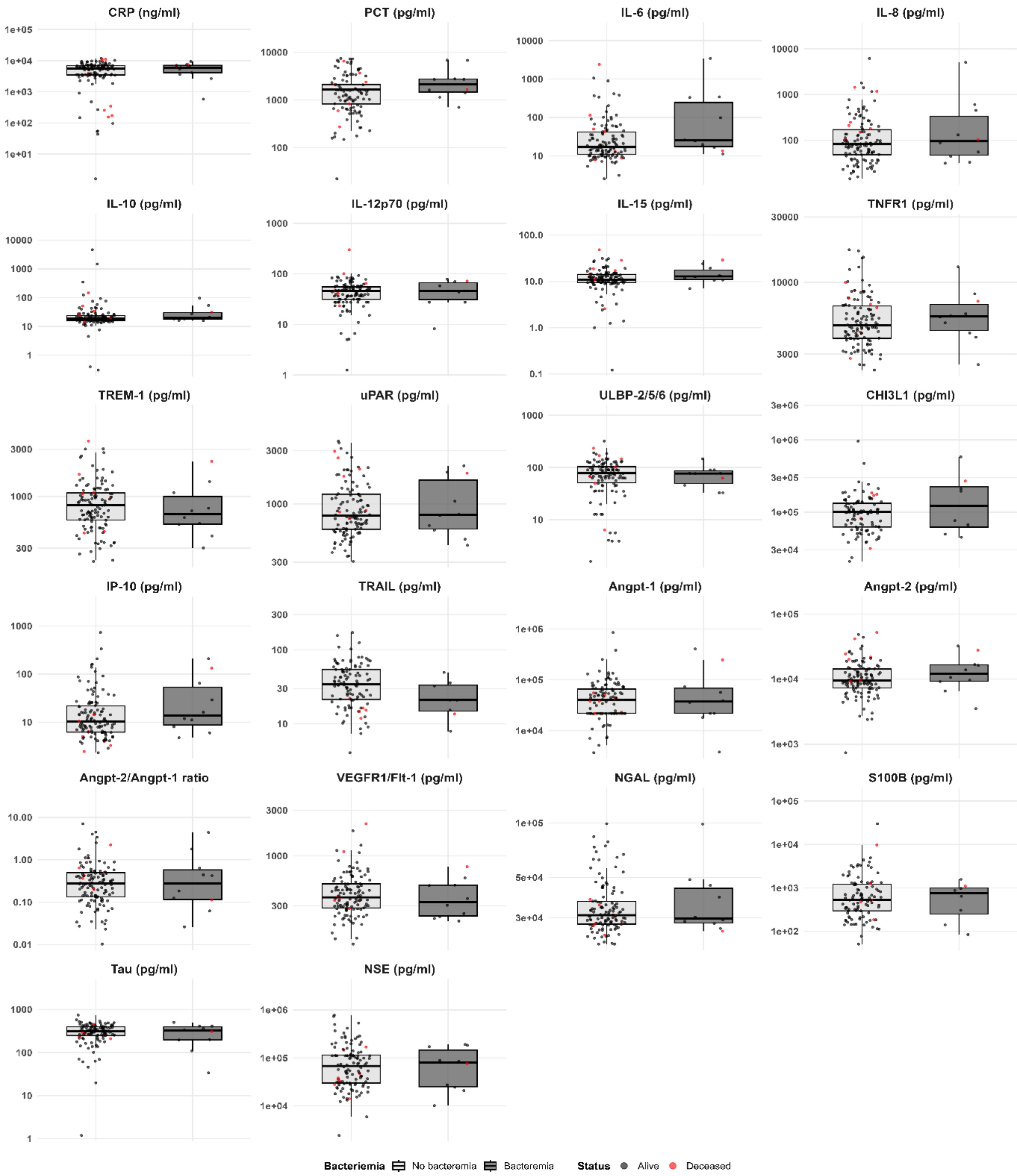

Legend: Boxplots showing the distribution of serum levels of immune and endothelial biomarkers of interest according to laboratory-confirmed bacterial sepsis: (i) patients without bacterial sepsis in light grey, and (ii) patients with laboratory-confirmed bacterial sepsis in dark grey.

Boxes represent the interquartile range (IQR), with horizontal lines indicating the median values. Each dot corresponds to an individual patient; and red dots indicate deceased patients.

Raw p-values were calculated using Mann–Whitney U tests. Statistically significant differences are indicated with asterisks: *: *p<0.05,* **: *p<0.01,* ***: *p<0.001*

Table S4. Incremental discrimination of selected biomarkers beyond clinical variables for prediction of in-hospital death

BIOMARKERS	AUROC clinical model (95% CI) [a]	AUROC clinical model corrected [b]	AUROC clinical model + biomarker (95% CI)[c]	AUROC clinical model + marker corrected [b]	Difference (95% CI)	Difference corrected [b]	p-value (LR test) [d]	n/events
**SERUM**								
**TRAIL**	0.776 (0.528-1.000)	0.716	0.902 (0.798-1.000)	0.849	0.126 (0.031 to 0.271)	0.133	0.018	91/6
**CSF**								
**PCT**	0.827 (0.682-0.971)	0.778	0.892 (0.791-0.993)	0.850	0.065 (0.012 to 0.176)	0.072	0.004	111/10

[a] Clinical model including age, sex, birth weight (continuous), and asphyxia.
[b] AUROCs and differences in AUROC were corrected for optimism using 1,000 bootstrap replications, with the full modelling procedures repeated in each bootstrap sample and performance evaluated in the original dataset.
[c] Clinical model including age, sex, birth weight (continuous), and asphyxia, and the biomarker.
[d] p-value from the likelihood ratio test comparing the clinical model with the corresponding clinical model plus biomarkers.
All AUROCs represent discrimination for in-hospital death. Models including each biomarker were fitted in the same complete-case sample for that biomarker.

Table S5. Distribution of CSF concentration of biomarkers according to in-hospital death

BIOMARKERS IN CSF (pg/ml) Median [IQR]	Survived (n=136)	Died (n=12)	p-value [a]	adjusted p-value [b]
**CRP (ng/ml)**	125.96 [57.90-284.97]	168.76 [106.46-341.57]	0.543	0.718
**PCT**	719.27 [561.34-1043.71]	1466.91 [1206.88-1861.40]	<0.0001	<0.0001
**IL-6**	3.33 [1.83-9.83]	15.45 [2.88-22.47]	0.139	0.341
**IL-8**	235.50 [164.68-483.06]	347.17 [158.12-1654.44]	0.393	0.646
**IL-10**	27.94 [19.27-50.25]	50.02 [24.15-103.55]	0.138	0.341
**IL-12p70**	17.10 [6.63-29.59]	18.92 [8.24-41.68]	0.261	0.521
**IL-15**	7.96 [6.58-9.82]	10.03 [6.92-14.20]	0.083	0.341
**TNFR1**	1724.02 [1429.02-2159.50]	2775.96 [1951.71-4881.75]	0.023	0.170
**TREM-1**	41.11 [28.59-68.56]	74.15 [43.22-320.72]	0.016	0.170
**uPAR**	184.17 [144.65-254.65]	285.55 [150.15-361.95]	0.096	0.341
**ULBP-2/5/6**	120.54 [88.20-173.49]	109.74 [86.53-197.50]	0.846	0.905
**CHI3L1**	48424.02 [31764.34-64429.18]	50446.33 [39558.36-83039.44]	0.555	0.718
**IP-10**	81.05 [42.17-163.45]	151.87 [60.11-449.12]	0.111	0.341
**TRAIL**	1.87 [0.94-4.07]	1.91 [0.94-3.29]	0.864	0.905
**Angpt-1**	80.91 [42.94-163.07]	111.91 [53.42-199.21]	0.703	0.814
**Angpt-2**	632.17 [427.30-982.04]	938.62 [412.80-1474.08]	0.411	0.646
**Angpt-2/ Angpt-1 ratio**	7.91 [4.10-14.15]	7.96 [6.57-19.99]	0.381	0.646
**VEGFR1/Flt-1**	139.50 [105.86-214.51]	107.89 [86.51-263.17]	0.528	0.718
**NGAL**	3350.96 [1927.10-6847.80]	3927.20 [1725.65-9244.09]	0.915	0.915
**Tau**	64.38 [44.56-96.04]	99.83 [66.11-212.58]	0.053	0.293
**S100B**	143.86 [47.65-427.73]	188.85 [54.29-564.92]	0.615	0.752
**NSE**	29091.42 [16183.82-50074.91]	42400.43 [19244.48-99130.09]	0.167	0.367

13 CSF samples with RBC >100,000 cells/µL were excluded from these analyses (10 from patients who survived and 3 from patients who died). Missing data for biomarkers assessment: CRP (n=0); PCT, TREM-1, TNFR1, Tau, IL-15, IL-6, NSE, uPAR, IL-12p70, IL-10, IL-8, IP-10, Angpt-2, Angpt-1, ULBP-2/5/6, NGAL, VEGFR1 (n=3 each: 3 survived and 0 died); TRAIL, CHI3L1, S100B (n = 12 each: 12 survived and 0 died)

[a] Unadjusted p-values calculated using the Wilcoxon rank-sum test.

[b] Adjusted p-values for multiple testing using the false discovery rate (FDR) correction across the full set of biomarkers.

Figure S3. Correlation of biomarker concentrations between paired serum and CSF samples

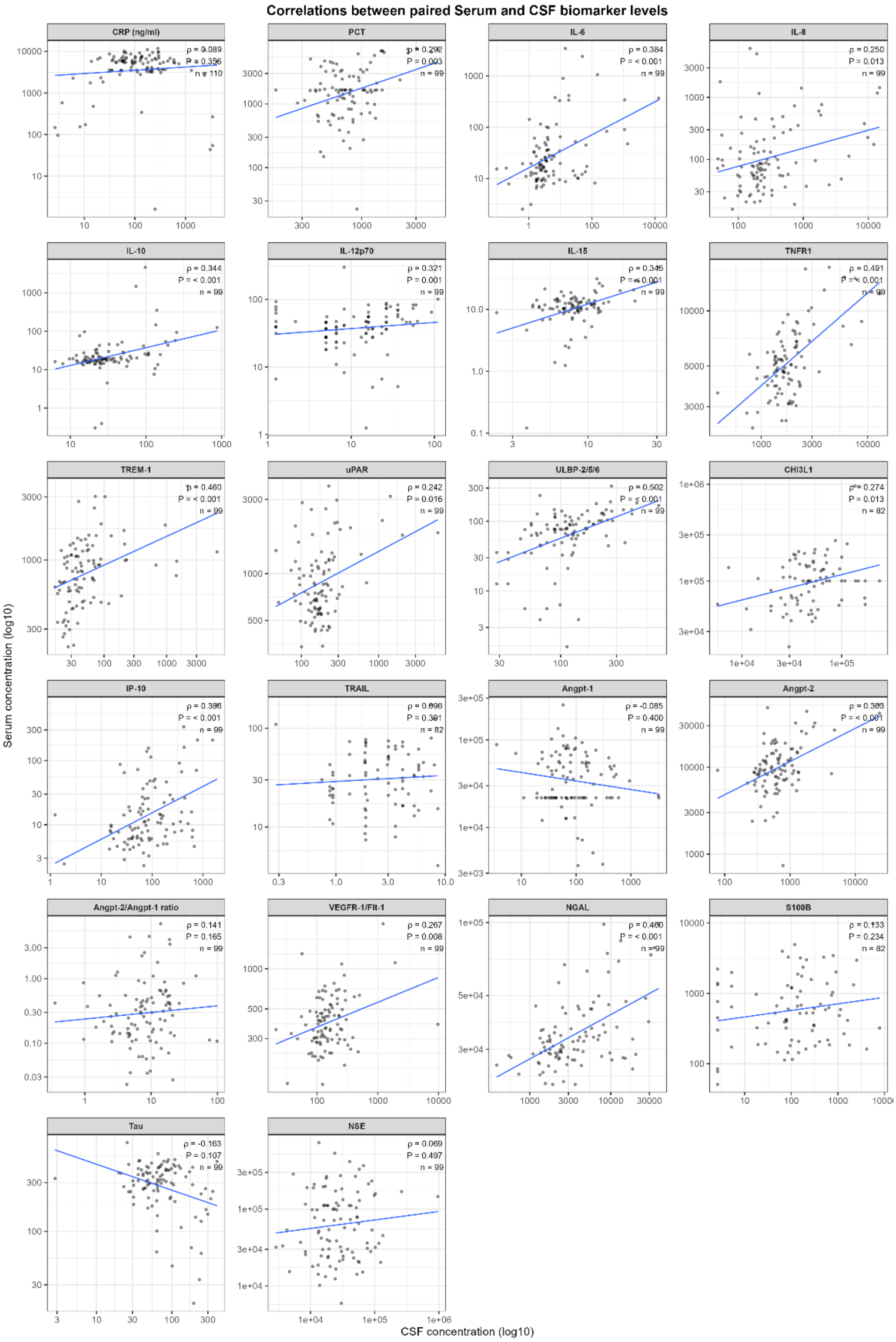

Correlations of paired levels of biomarkers measured in serum and CSF. ρ (rho) and *p*-values were calculated by Spearman and are indicated in the figure.

8 CSF samples with RBC >100,000 cells/µL were excluded, resulting in 110 paired samples available for analysis. Missing data for biomarkers assessment: CRP (n=0); PCT, TREM-1, TNFR1, Tau, IL-15, IL-6, NSE, uPAR, IL-12p70, IL-10, IL-8, IP-10, Angpt-2, Angpt-1, ULBP-2/5/6, NGAL, VEGFR1 (n=11 each); TRAIL, CHI3L1, S100B (n = 28 each).

Table S6. AUROC of selected serum and CSF biomarkers for predicting in-hospital death in paired samples

Biomarkers	Serum AUROC (95% CI)	CSF AUROC (95% CI)	Combined AUROC (95% CI)
**IL-8**	0.81 (0.70-0.93)	0.49 (0.15-0.83)	0.56 (0.26-0.87)
**uPAR**	0.74 (0.56-0.92)	0.53 (0.16-0.90)	0.74 (0.54-0.94)
**TRAIL**	0.85 (0.75-0.95)	0.55 (0.14-0.96)	0.85 (0.75-0.95)
**Angpt-2**	0.82 (0.64-0.99)	0.57 (0.19-0.95)	0.73 (0.45-1.00)
**PCT**	0.55 (0.25-0.84)	0.85 (0.73-0.98)	0.85 (0.75-0.95)

AUROC for in-hospital death of serum and CSF biomarkers in paired samples. 8 CSF samples with RBC >100,000 cells/µL were excluded, resulting in 110 paired samples available for analysis, including 9 deaths. Missing data for biomarkers assessment: uPAR, IL-8, Angpt-2, PCT (n=11 each; 3 deaths); TRAIL (n = 28; 5 deaths).

Table S7. Distribution of serum concentrations of biomarkers according to the diagnosis of probable or laboratory-confirmed meningitis

BIOMARKERS IN SERUM (pg/ml) Median [IQR]	Probable or laboratory-confirmed meningitis (n=33)*	No meningitis (n=103)*	p-value [a]	adjusted p-value [b]
**CRP (ng/ml)**	5379.33 [3505.71-6813.03]	5853.13 [3558.08-6967.47]	0.399	1.000
**PCT**	1650[1144.68-2700.11]	1628.47 [713.33-2104.00]	0.212	1.000
**IL-6**	25.45 [11.47-63.46]	16.85 [10.63-35.94]	0.141	1.000
**IL-8**	88.84 [55.32-221.15]	79.29 [38.13-152.71]	0.239	1.000
**IL-10**	19.68 [16.48-28.38]	18.72 [16.12-23.01]	0.242	1.000
**IL-12p70**	47.17 [39.33-55.49]	39.33 [27.53-55.49]	0.103	1.000
**IL-15**	12.39 [9.68-14.17]	10.98 [9.38-14.18]	0.382	1.000
**TNFR1**	5080.77 [4054.51-6541.68]	4908.65 [3911.79-6795.64]	0.830	1.000
**TREM-1**	686.81 [520.44-1014.92]	821.36 [577.42-1128.93]	0.383	1.000
**uPAR**	743.79 [546.52-1224.24]	798.73 [604.76-1224.22]	0.370	1.000
**ULBP-2/5/6**	67.82 [49.52-90.67]	77.48 [56.20-107.04]	0.366	1.000
**CHI3L1**	101994.73 [60617.35-153212.86]	100052.52 [62689.33-131377.06]	0.402	1.000
**IP-10**	15.78 [6.19-51.39]	9.84 [6.31-18.00]	0.151	1.000
**TRAIL**	32.83 [17.59-46.74]	34.36 [20.96-55.97]	0.570	1.000
**Angpt-1**	38612.78 [21850.00-56584.21]	40062.27 [21850.00-71214.13]	0.490	1.000
**Angpt-2**	10475.37 [8490.53-14693.93]	9538.12 [7160.53-14432.40]	0.442	1.000
**Angpt-2/Angpt-1 ratio**	0.27 [0.13-0.63]	0.28 [0.12-0.47]	0.378	1.000
**VEGFR1/Flt-1**	383.50 [252.53-495.81]	353.48 [286.86-511.05]	0.894	1.000
**NGAL**	29004.74 [27522.10-36615.43]	31986.55 [27860.02-37524.12]	0.154	1.000
**S100B**	665.69 [300.98-1156.99]	521.75 [274.19-1204.42]	0.440	1.000
**Tau**	283.84 [208.32-400.68]	316.51 [252.97-388.08]	0.457	1.000
**NSE**	75665.95 [29987.42-183266.11]	71323.70 [29441.05-111010.00]	0.513	1.000

Missing data for biomarkers assessment: CRP (n=0); PCT, TREM-1, TNFR1, Tau, IL-15, IL-6, NSE, uPAR, IL-12p70, IL-10, IL-8, IP-10, Angpt-2, Angpt-1, ULBP-2/5/6, NGAL, VEGFR1 (n=8 each); TRAIL, CHI3L1, S100B (n = 24 each)

[a] p-values computed using Mann-Whitney U tests.

[b] p-values computed using Mann–Whitney U tests, adjusted for multiple comparisons using the Holm method.

Probable or laboratory-confirmed meningitis defined as CSF pleocytosis ≥30 white blood cells (WBC)/mm3 and/or positive culture or PCR

Table S8. Distribution of CSF concentrations of biomarkers according to the diagnosis of probable or laboratory-confirmed meningitis

BIOMARKERS IN CSF (pg/ml) Median [IQR]	Probable or laboratory-confirmed meningitis (n=36)	No meningitis (n=112)	p-value [a]	adjusted p-value [b]
**CRP (ng/ml)**	181.46 [64.96-429.88]	122.30 [57.9-251.55]	0.108	1.000
**PCT**	785.73 [588.52-1228.07]	754.6 [569.89-1143.32]	0.906	1.000
**IL-6**	6.34 [3.25-23.27]	2.95 [1.77-9.46]	0.006	0.130
**IL-8**	267.31 [192.80-1208.16]	229.74 [155.72-473.38]	0.081	1.000
**IL-10**	37.58 [19.96-147.26]	26.91 [20.89-47.21]	0.129	1.000
**IL-12p70**	29.59 [8.24-36.75]	16.12 [6.63-29.59]	0.016	0.280
**IL-15**	8.53 [6.78-11.81]	7.78 [6.71-9.82]	0.140	1.000
**TNFR1**	2062.7 [1564.3-2477.8]	1670.22 [1409.07-2159.06]	0.043	0.645
**TREM-1**	65.13 [36.34-128.74]	39.24 [28.69-65.72]	0.016	0.280
**uPAR**	196.25 [164.17-259.11]	178.78 [143.33-266.21]	0.281	1.000
**ULBP-2/5/6**	124.43 [88.2-181.61]	116.61 [83.17-174.85]	0.467	1.000
**CHI3L1**	53065.66 [39589.73-96466.54]	46810.77 [31114.1-62410.76]	0.066	1.000
**IP-10**	114.77 [50.77-380]	75.75 [41.94-153.82]	0.090	0.990
**TRAIL**	3.02 [0.94-4.34]	1.87 [0.94-3.5]	0.296	1.000
**Angpt-1**	136.68 [57.94-286.05]	79.06 [41.85-150.24]	0.014	0.274
**Angpt-2**	709.16 [391.73-1082.69]	634.24 [450.72-1035.29]	0.585	1.000
**Angpt-2/Angpt-1 ratio**	4.8 [2.61-8.83]	8.76 [4.85-15.24]	0.003	0.061
**VEGFR1/Flt-1**	138.53 [107.17-244.18]	136.01 [102.12-213.64]	0.373	1.000
**NGAL**	6143.21 [2180.67–17737.53]	2898.30 [1783.90–5183.15]	0.006	0.117
**S100B**	178.43 [25.78-555.82]	144.12 [56.58-404.79]	0.891	1.000
**Tau**	70.16 [47.91-231.79]	64.38 [42.79-94.4]	0.101	1.000
**NSE**	46888.67 [19382.99-78215.91]	28396.15 [16035.57-43830.74]	0.019	0.302

13 CSF samples with RBC >100,000 cells/µL were excluded from these analyses (5 from participants with probable and laboratory-confirmed meningitis and 8 from participants with no meningitis). Missing data for biomarkers assessment: CRP (n=0); PCT, TREM-1, TNFR1, Tau, IL-15, IL-6, NSE, uPAR, IL-12p70, IL-10, IL-8, IP-10, Angpt-2, Angpt-1, ULBP-2/5/6, NGAL, VEGFR1 (n=3 each; 3 in non-meningitis group); TRAIL, CHI3L1, S100B (n = 12 each; 5 in meningitis and 7 in non-meningitis group)

[a] p-values computed using Mann-Whitney U tests.

[b] p-values computed using Mann–Whitney U tests, adjusted for multiple comparisons using the Holm method.

Probable or laboratory-confirmed meningitis was defined as CSF pleocytosis ≥30 white blood cells (WBC)/mm3 and/or positive culture or PCR.

Table S9. Associations between CSF biomarker concentrations and probable or laboratory-confirmed meningitis in the CSF study population

	Crude [a]		Adj. by sex and age [b]		Adj. by sex, age, and very low birth weight [c]	
	OR (95% CI)	p-value	aOR1 (95% CI)		aOR2 (95% CI)	p-value
**IL-6**	1.29 (1.10-1.54)	0.002	1.30 (1.11-1.55)	0.002	1.30 (1.11-1.55)	0.002
**IL-12p70**	1.38 (1.01-1.94)	0.052	1.41 (1.03-2.01)	0.042	1.40 (1.02-1.99)	0.049
**TNFR1**	1.85 (1.10-3.20)	0.022	1.95 (1.15-3.39)	0.015	1.92 (1.12-3.41)	0.020
**TREM-1**	1.44 (1.13-1.89)	0.005	1.46 (1.14-1.92)	0.004	1.45 (1.13-1.91)	0.005
**Angpt-1**	1.43 (1.12-1.87)	0.005	1.42 (1.11-1.88)	0.007	1.41 (1.10-1.85)	0.008
**Angpt-2/ Angpt-1 ratio**	0.55 (0.37-0.80)	0.002	0.56 (0.37-0.81)	0.004	0.56 (0.37-0.81)	0.004
**NGAL**	1.66 (1.23-2.27)	0.001	1.67 (1.24-2.30)	0.001	1.68 (1.24-2.34)	0.001
**NSE**	1.44 (1.06-2.01)	0.023	1.46 (1.07-2.04)	0.020	1.44 (1.06-2.02)	0.023

[a] ORs, 95% CI and p-values were obtained from univariable logistic regression models.
[b] ORs, 95% CI and p-values were obtained from multivariable logistic regression models adjusting for age, and sex.
[c] ORs, 95% CI and p-values were obtained from multivariable logistic regression models adjusting for age, sex, and very low birth weight.
All ORs indicate the increase in odds of the outcome for a two-fold increase in each biomarker concentration.
Probable or laboratory-confirmed meningitis was defined as CSF pleocytosis ≥30 white blood cells (WBC)/mm3 and/or positive culture or PCR.
13 CSF samples with RBC >100,000 cells/µL were excluded from these analyses (5 from participants with probable and laboratory-confirmed meningitis and 8 from participants with no meningitis). Missing data for biomarkers assessment: all markers (n=3 each; 3 in non-meningitis group).

Table S10. Distribution of CSF concentrations of biomarkers according to the diagnosis of microbiologically confirmed bacterial meningitis

BIOMARKERS IN CSF (pg/ml) Median [IQR]	Microbiologically confirmed bacterial meningitis (n=16)*	No Meningitis (n=112)	p-value [a]	adjusted p-value [b]
**CRP (ng/ml)**	170.66 [53.98-365.87]	122.30 [57.90-251.55]	0.533	1.000
**PCT**	694.71 [499.97-867.22]	754.60 [569.89-1143.32]	0.220	1.000
**IL-6**	4.19 [3.51-21.11]	2.95 [1.77-9.46]	0.019	0.425
**IL-8**	314.18 [192.51-1148.21]	229.74 [155.72-473.38]	0.202	1.000
**IL-10**	32.02 [19.53-68.11]	26.91 [20.89-47.21]	0.460	1.000
**IL-12p70**	17.10 [5.08-29.59]	16.12 [6.63-29.59]	0.694	1.000
**IL-15**	7.99 [6.72-11.29]	7.78 [6.71-9.82]	0.508	1.000
**TNFR1**	1810.35 [1535.34-2594.84]	1670.22 [1409.07-2159.06]	0.210	1.000
**TREM-1**	43.46 [29.59-133.58]	39.24 [28.69-65.72]	0.282	1.000
**uPAR**	189.10 [134.16-259.58]	178.78 [143.33-266.21]	0.836	1.000
**ULBP-2/5/6**	108.28 [88.20-138.07]	116.61 [83.17-174.85]	0.862	1.000
**CHI3L1**	50672.66 [44518.47-56819.47]	46810.77 [31114.10-62410.76]	0.495	1.000
**IP-10**	110.95 [49.53-216.96]	75.75 [41.94-153.82]	0.313	1.000
**TRAIL**	3.50 [0.94-4.07]	1.87 [0.94-3.50]	0.419	1.000
**Angpt-1**	89.78 [46.89-203.11]	79.06 [41.85-150.24]	0.381	1.000
**Angpt-2**	560.03 [354.46-1108.73]	634.24 [450.72-1035.29]	0.587	1.000
**Angpt-2/Angpt-1 ratio**	6.28 [3.73-10.75]	8.76 [4.85-15.24]	0.133	1.000
**VEGFR1/Flt-1**	137.66 [124.02-271.23]	136.01 [102.12-213.64]	0.197	1.000
**NGAL**	3714.13 [2085.34-8425.99]	2898.30 [1783.90-5183.15]	0.389	1.000
**S100B**	268.41 [47.32-627.59]	144.12 [56.58-404.79]	0.592	1.000
**Tau**	67.87 [42.49-95.46]	64.38 [42.79-94.40]	0.567	1.000
**NSE**	28021.97 [18212.21-61120.17]	28396.15 [16035.57-43830.74]	0.418	1.000

13 CSF samples with RBC >100,000 cells/µL were excluded from these analyses. Missing data for biomarkers assessment: CRP (n=0); PCT, TREM-1, TNFR1, Tau, IL-15, IL-6, NSE, uPAR, IL-12p70, IL-10, IL-8, IP-10, Angpt-2, Angpt-1, ULBP-2/5/6, NGAL, VEGFR1 (n=3 each; 3 in the non-meningitis group); TRAIL, CHI3L1, S100B (n = 8 each; 1 in the meningitis and 7 in the non-meningitis group)
Microbiologically confirmed bacterial meningitis was defined as identification of a bacterial pathogen in CSF by culture or PCR.
[a] p-values computed using Mann-Whitney U tests.
[b] p-values computed using Mann–Whitney U tests, adjusted for multiple comparisons using the Holm method.

Table S11. Associations between CSF biomarker concentrations and microbiologically confirmed bacterial meningitis in the CSF study population

	Crude [a]		Adj. by sex and age [b]		Adj. by sex, age, and very low birth weight [c]	
	**OR (95% CI)**	**p-value**	**aOR1 (95% CI)**	**p-value**	**aOR2 (95% CI)**	**p-value**
**IL-6**	1.32 (1.08-1.64)	0.007	1.34 (1.09-1.67)	0.006	1.35 (1.10-1.69)	0.005

[a] ORs, 95% CI and p-values were obtained from univariable logistic regression models.
[b] ORs, 95% CI and p-values were obtained from multivariable logistic regression models adjusting for age, and sex.
[c] ORs, 95% CI and p-values were obtained from multivariable logistic regression models adjusting for age, sex, and very low birth weight.
All ORs indicate the increase in odds of the outcome for a two-fold increase in each biomarker concentration.
Microbiologically confirmed bacterial meningitis was defined as identification of a bacterial pathogen in CSF by culture or PCR.
13 CSF samples with RBC >100,000 cells/μL were excluded from these analyses. Missing data for biomarkers assessment: IL-6 (n=3; 3 in the non-meningitis group).

Table S12. Distribution of CSF biomarker concentrations according to in-hospital death in patients with probable or laboratory-confirmed meningitis

BIOMARKERS IN CSF (pg/ml) Median [IQR]	Probable or laboratory-confirmed meningitis (n=36)*		p-value [a]	adjusted p-value [b]
	Survived (n=33)	Died (n=3)		
**CRP (ng/ml)**	188.50 [68.04-408.47]	135.97 [84.58-325.49]	0.689	1.000
**PCT**	781.49 [544.78-1027.09]	2132.00 [1680.03-2152.72]	0.022	0.490
**IL-6**	5.29 [3.27-26.06]	14.61 [8.07-18.94]	1.000	1.000
**IL-8**	281.72 [193.83-1152.69]	249.24 [162.84-7015.19]	0.925	1.000
**IL-10**	35.79 [18.66-138.99]	51.82 [39.37-99.54]	0.594	1.000
**IL-12p70**	26.41 [8.24-36.75]	40.32 [34.96-66.24]	0.103	1.000
**IL-15**	8.42 [6.82-11.03]	13.03 [9.87-17.71]	0.332	1.000
**TNFR1**	1883.77 [1555.12-2450.58]	4167.27 [3166.48-5596.23]	0.074	1.000
**TREM-1**	63.17 [31.51-108.69]	128.74 [86.74-788.89]	0.222	1.000
**uPAR**	188.88 [153.85-252.86]	459.65 [359.38-578.63]	0.042	0.878
**ULBP-2/5/6**	124.43 [88.52-169.24]	181.61 [128.99-213.39]	0.684	1.000
**CHI3L1**	53795.81 [37426.34-95969.37]	45067.36 [43154.25-72593.68]	0.894	1.000
**IP-10**	110.95 [47.04-380.00]	136.85 [111.61-356.21]	0.471	1.000
**TRAIL**	3.02 [0.94-4.21]	0.94 [0.94-4.75]	0.919	1.000
**Angpt-1**	129.64 [55.17-264.42]	246.21 [172.67-1719.66]	0.301	1.000
**Angpt-2**	646.12 [393.09-1060.06]	860.07 [549.66-11990.28]	0.639	1.000
**Angpt-2/Angpt-1 ratio**	4.80 [2.62-9.10]	7.24 [4.11-7.96]	0.876	1.000
**VEGFR-1/Flt-1**	140.26 [114.64-238.81]	93.55 [74.21-658.50]	0.434	1.000
**NGAL**	6138.46 [2205.35-15743.68]	14156.64 [8079.87-20938.68]	0.552	1.000
**S100B**	210.22 [29.01-540.82]	47.12 [26.18-2842.87]	0.973	1.000
**Tau**	67.87 [46.85-221.07]	94.36 [80.33-168.40]	0.491	1.000
**NSE**	46074.21 [20410.67-76629.29]	49308.80 [33295.24-155532.08]	0.639	1.000

Probable or laboratory-confirmed meningitis was defined as CSF pleocytosis ≥30 white blood cells (WBC)/mm3 and/or positive culture or PCR.
*5 CSF samples with RBC >100,000 cells/µL were excluded from these analyses.
[a] p-values computed using Mann-Whitney U tests.
[b] p-values computed using Mann–Whitney U tests, adjusted for multiple comparisons using the Holm method.
13 CSF samples with RBC >100,000 cells/µL were excluded from these analyses. Missing data for biomarkers assessment: CRP (n=0); PCT, TREM-1, TNFR1, Tau, IL-15, IL-6, NSE, uPAR, IL-12p70, IL-10, IL-8, IP-10, Angpt-2, Angpt-1, ULBP-2/5/6, NGAL, VEGFR1 (n=3 each); TRAIL, CHI3L1, S100B (n = 5 each)
